# Sonographic Aeration Scoring Indicates Disease Severity in Critically Ill Patients with COVID-19

**DOI:** 10.3390/diagnostics13223446

**Published:** 2023-11-15

**Authors:** Daniel T. Marggrander, Philippe Simon, Tobias Schröder, Daniel Gill-Schuster, Haitham Mutlak

**Affiliations:** 1 Department of Anaesthesiology, Intensive Care and Pain Therapy, Sana Hospital Offenbach, 63069 Offenbach am Main, Germany; 2 Department of Interdisciplinary Emergency Medicine, Sana Hospital Offenbach, 63069 Offenbach am Main, Germany; 3 Department of Anaesthesiology, Intensive Care and Pain Therapy, University Hospital Frankfurt, 60596 Frankfurt am Main, Germany

**Keywords:** SARS-CoV-2, lung ultrasound, ARDS, ECMO

## Abstract

Aims and Methods: We evaluated an ultrasound score from 0 to 32 points in eight pulmonary regions to monitor critically ill COVID-19 patients. The score was correlated to surrogate parameters of disease severity, i.e., the oxygenation index, respiratory support, mortality, plasma interleukin-6, and WHO and ARDS classifications. Results: A total of 27 patients were repeatedly examined, and 71 examinations were evaluated. Patients with severe COVID-19 scored higher (median 17) than those with moderate disease (median 11, *p* < 0.01). The score did not differentiate between stages of ARDS as defined by the Berlin criteria (*p* = 0.1) but could discern ARDS according to the revised ESICM definition (*p* = 0.002). Non-survivors had higher ultrasound scores than survivors (median 18.5 vs. 14, *p* = 0.04). The score correlated to the oxygenation index (ρ = −0.56, *p* = 0.03), and changes in the score between examinations correlated to changes in oxygenation (ρ = −0.41, *p* = 0.16). The correlation between the score and interleukin-6 was ρ = 0.35 (*p* < 0.001). The interrater reliability for the score was ICC = 0.87 (*p* < 0.001). Conclusions: The ultrasound score is a reliable tool that might help monitor disease severity and may help stratify the risk of mortality.

## 1. Introduction

**Figure 1:**
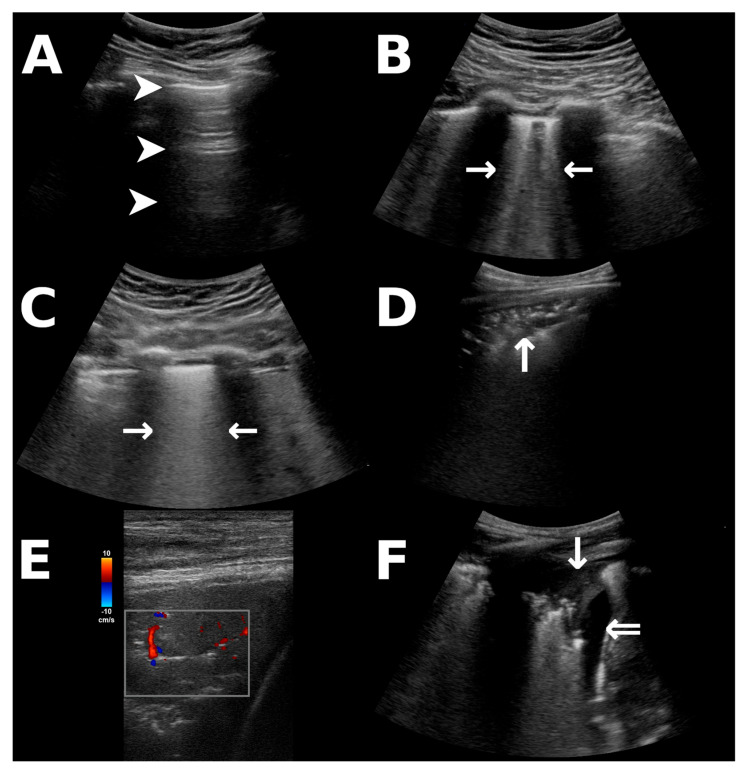
Ultrasonographic findings that were incorporated into the scoring system. All images were obtained from patients in our study population. (**A**) A-lines as reverberations of the pleural line (arrowheads) indicate fully aerated lungs or pneumothorax, distinguishable by dynamic pleural gliding [3,4]. (**B**,**C**) B-lines (horizontal arrows) arising from the pleural line indicate interstitial pathology and appear distinct (**B**) or merge to form coalescent B-lines (**C**), the latter corresponding to alveolar edema [4]. (**D**) Peripheral consolidations (vertical arrow) appear tissue-like and may contain hyperechoic air bronchograms. (**E**) Color Doppler ultrasonography of a pulmonary consolidation (red: flow towards the ultrasound probe, blue: flow away from the probe; scale for reference) in COVID-19 may demonstrate diminished pulmonary perfusion [1]. (**F**) Atelectatic lung (vertical arrow) within a pleural effusion (double arrow).

During the ongoing pandemic of coronavirus disease-19 (COVID-19), lung ultrasound (LUS) has emerged as a powerful diagnostic tool in detecting the pulmonary involvement of infection [1]. LUS provides bedside imaging without radiation exposure and can be repeated perpetually while providing a higher sensitivity than bedside radiography, particularly in COVID-19 [1,2]. Interstitial involvement in lung disease is indicated by B-lines on LUS [3,4], which are distinguishable in moderate pathologies (Figure 1B) and may form indistinguishable, coalescent B-lines (Figure 1C) when interstitial involvement progresses to alveolar edema, corresponding to “white lung” in conventional imaging [4]. B-lines may occur in infectious disease, pulmonary edema, and other interstitial lung diseases [3,4]. Furthermore, consolidations that reach the pleura and pleural effusion (Figure 1D–F) are detectable via LUS [1,3]. Critically ill patients are at a high risk of suffering from complications during in-hospital transport to acquire radiological imaging [5], whereas LUS can be performed at the patients’ bedside. Many pulmonary complications in the intensive care unit (ICU) unrelated to COVID-19, for instance, the development of a pneumothorax, are also reliably detected via LUS [4].

Severe cases of COVID-19 can result in acute respiratory distress syndrome (ARDS), requiring mechanical ventilation or extracorporeal membrane oxygenation (ECMO) therapy. Before the beginning of the COVID-19 pandemic, lung ultrasound scoring systems were evaluated for predicting disease severity in ARDS [6] and extubation success in mechanically ventilated patients [7] or to monitor patients requiring ECMO [8]. To assess the sonographic scoring system for monitoring COVID-19 over time, we performed a prospective study, correlating a LUS score in critically ill patients with COVID-19 to their disease severity.

## 2. Materials and Methods

We consecutively enrolled patients in the anesthesiologic ICU at Sana Hospital Offenbach, Germany, who tested positive for SARS-CoV-2 in a polymerase chain reaction (PCR) test of a nasopharyngeal swab. We excluded patients who were not examined via LUS within 5 days after admission. All patients or their legal guardians gave informed consent.

**Figure 2:**
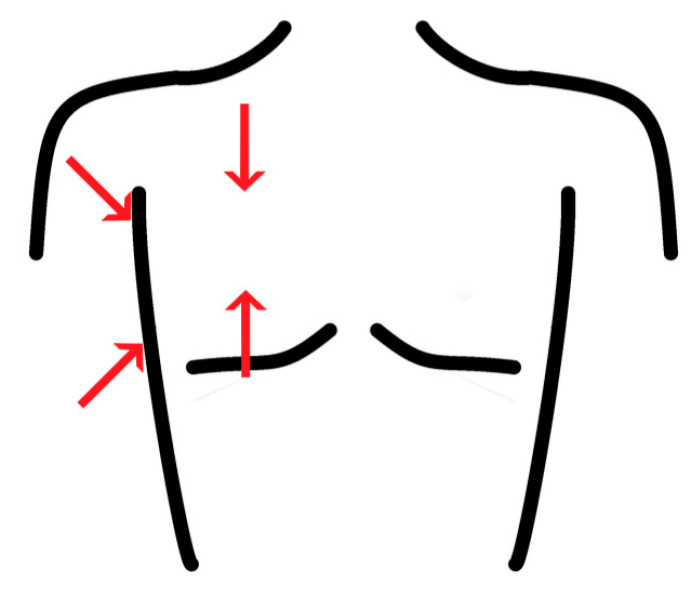
Schematic sketch of the LUS areas assessed per hemithorax.

LUS was performed repeatedly every two days by one of two examiners (D.T.M. or P.S.). If the examination interfered with proning schedules, follow-up examinations were performed as soon as possible, either before or after the two-day interval. Using a Vivid iq (GE Healthcare, Chicago, IL, USA) or a Sonosite X-Porte (Fujifilm Sonosite, Bothell, WA, USA) ultrasound device with a convex probe, we performed two anterior scans per the midclavicular line (2nd and 5th intercostal space, ICS) and two per posterior axillary line (3rd and 6th ICS, Figure 2). This was performed in accordance with evidence-based guidelines recommending scans of eight regions in point-of-care LUS [9]. Imaging artifacts were noted, scoring 0 points for A-lines (Figure 1A), 1 point for three or more B-lines per ICS (Figure 1B), 2 points for coalescent B-lines (Figure 1C), 3 points for consolidations (Figure 1D), and 4 points for an atelectatic lung within a pleural effusion (Figure 1F). The score could therefore range between 0 and 32 points. This scoring system reflects aeration of pulmonary tissue, ranging from a completely aerated lung (when A-lines are present) to complete absence of aeration (when the pleural cavity is occupied by effusion), with each additional point reflecting a decrease in the air/fluid ratio of the lung tissue adjacent to the pleura [4]. We selected this score from previous studies investigating ARDS [6], as we expected to enroll patients with severe respiratory distress; however, we decided to add the category of pleural effusion with an atelectatic lung analogous to other scoring systems [7] as it reflects further deterioration of aeration compared to consolidation alone [4]. The recorded images were also scored by the other examiner, blinded to the clinical presentation for the assessment of interrater reliability (see below).

The arterial blood gas (BGA), oxygenation index (p_a_O_2_/F_i_O_2_), and respiratory support at the time of the examination were documented. Furthermore, plasma interleukin-6 (IL-6) levels and the treatment with dexamethasone and tocilizumab were noted as markers of disease severity. Other pulmonary infections were diagnosed through cultures or PCRs from obtained sputum or bronchial lavage. All invasive procedures were performed as part of regular patient care. The arterial BGA was recorded routinely three times daily or more frequently as required, and the IL-6 levels were tested daily. The most current BGA level that was obtained under the same respiratory support was used for this study. Inspiratory oxygen (F_i_O_2_) was noted in patients receiving high-flow oxygen (HFOT), invasive or non-invasive (NIV) ventilation, or ECMO therapy, and was estimated in patients receiving low-flow oxygen (LFOT) over nasal cannulas or masks. In NIV and invasive ventilation, positive end-expiratory pressure (PEEP) was documented. All treatment decisions were part of ongoing patient care, in which neither D.T.M. nor P.S. was involved. At each examination, the criteria of the Berlin classification for ARDS and the World Health Organization (WHO) classification for the disease severity of COVID-19 were applied [10,11]. Considering the amendment of the European Society of Intensive Care Medicine (ESICM) to the ARDS definition in 2023 [12], we included the renewed ARDS guidelines post hoc as well. The mortality was surveyed until patients were discharged or referred from our hospital. The trial received approval from the ethics committee of the Chamber of Physicians of Hesse (2021-2478-evBO).

The normal variables are depicted as the mean ± standard deviation (SD), while the ordinal or non-normally distributed variables are depicted as the median with interquartile range (IQR). The frequencies are noted as a percentage. The continuous variables were compared using either Student’s two-sided t-test, an analysis of variance (ANOVA), the Wilcoxon–Mann–Whitney test, or the Kruskal–Wallis test depending on normality, which was tested using the Shapiro–Wilk test. Polyserial correlation (ρ) was used to correlate ordinal to continuous variables. Cutoff points of the LUS scores were based on receiver operating characteristic (ROC) curves, reporting the corresponding area under the curve (AUC), sensitivity (Sn), and specificity (Sp). Interrater reliability between the two observers was calculated using a two-sided intraclass correlation (ICC) between the score of the primary examiner and the score based on the blinded assessment of the same images by the other examiner. We used GNU R 4.1.1 for analysis [13].

## 3. Results

**Figure 3:**
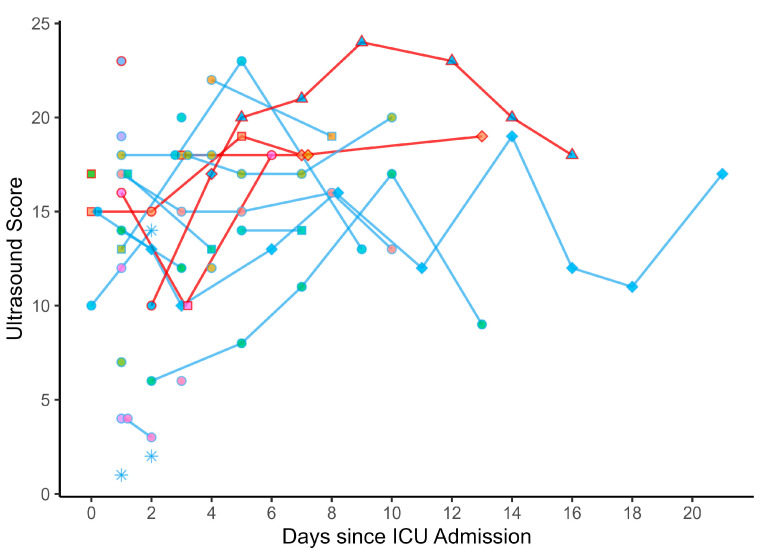
LUS scores of the patients over the course of the study. Patients who died are outlined in dark red and survivors are outlined in blue. The fill of each dot corresponds to an individual patient to avoid confusion in the overlapping plots. Asterisk: no respiratory support. Circle: supplemental oxygen (LFOT or HFOT). Squares: NIV. Diamonds: invasive ventilation. Triangles: ECMO therapy.

Seventy-one LUS examinations were performed on 27 individual patients between November 2021 and March 2022, and their mean age was 55.6 years ± 17.3. Eighteen patients (66.7%) were male. The median score at the initial examination was 14 (IQR 8.5–17.5) and the highest score per patient was a median of 17 (IQR 12–19). The initial exam was performed a median of one day after admission (IQR 1–2.5), and follow-up examinations were performed a median of every two days (IQR 2–3). For the detailed scores and timeframe, please see Figure 3. According to the WHO classification, in 50 instances (70.4%) we witnessed severe COVID-19, while 21 examinations (29.6%) were performed for moderate disease. In severe disease, the patients scored a median of 17 (IQR 14–18) on LUS, compared to a median of 11 (IQR 6–14) in those with moderate COVID-19 at the time of examination (*p* < 0.001). A cutoff score of 14 distinguished between moderate and severe COVID-19 in this population, with Sn = 0.76 and Sp = 0.71 (AUC = 0.75).

**Figure 4:**
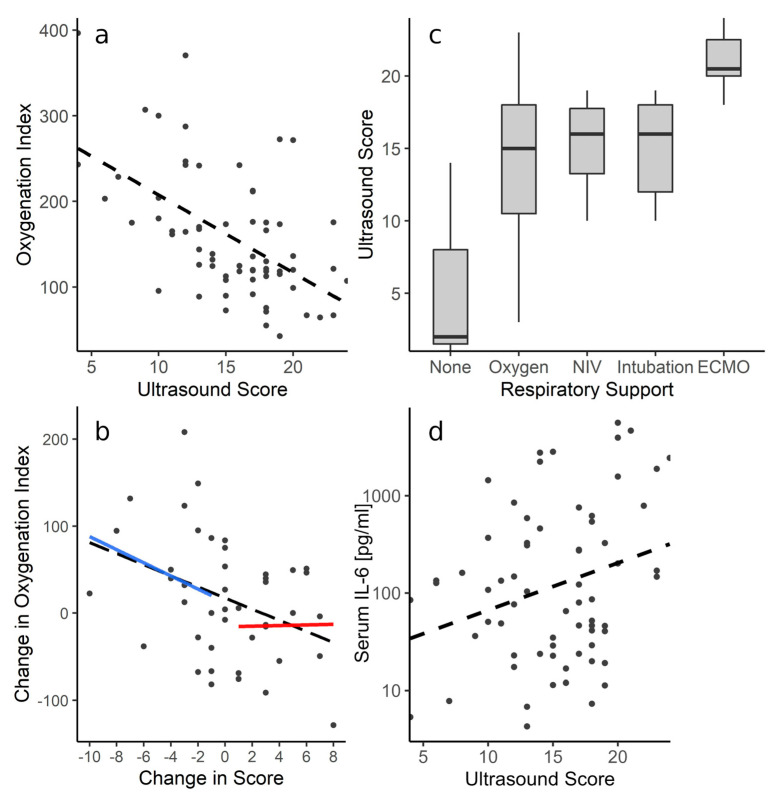
(**a**) LUS score versus corresponding oxygenation (or Horovitz) index, ρ = −0.56. (**b**) Between follow-up examinations, the change in LUS score correlates to changes in oxygenation (ρ = −0.41), particularly when the score decreases (ρ = −0.35, blue line). In contrast, an increase in the score barely correlates to a change in oxygenation (ρ = 0.04, red line) between follow-up examinations. (**c**) LUS score according to disease severity, as indicated by required respiratory support (*p* < 0.01, Kruskal–Wallis test). (**d**) Correlation of the score and serum IL-6 levels (ρ = 0.35).

The median oxygenation index at the initial examination was 155.9 (IQR 117.6–242.3) and the lowest oxygenation index per patient was a median of 71.5 (IQR 62.8–83.5). The LUS score showed a negative correlation with the oxygenation index (ρ = −0.56, *p* = 0.03, Figure 4a) and the changes in score correlated inversely to changes in oxygenation (ρ = −0.41, *p* = 0.16, Figure 4b). When the score decreased between examinations, its correlation to changes in oxygenation was stronger (ρ = −0.35, *p* = 0.1) compared to when the score increased (ρ = 0.04, *p* = 0.26).

Six patients (22.2%) died and twenty-one (77.8%) were referred alive. The median peak score per patient in non-survivors was 18.5 (IQR 18–22), versus a median of 14 (IQR 7–19) in survivors (*p* = 0.04). Calculating a cutoff of 18 for the highest score per patient (AUC = 0.79) yielded Sn = 0.83 and Sp = 0.67 for a fatal outcome.

Superinfections were observed in twenty-nine instances among the 71 examinations (40.8%): bacterial superinfection coincided in twelve, *Aspergillus* in two, and a combined bacterial and fungal superinfection in fifteen instances, resulting in higher scores (median 17, IQR 14–19) than in patients without superinfection (median 14, IQR 10–17, *p* = 0.01).

In three of the seventy-one examinations (4.2%), the patients required no respiratory support (median score 2, IQR 1.5–8), and in thirty-nine (54.9%), they required either LFOT or HFOT (median score 15, IQR 10.5–18). In ten (14.1%) examinations, the patients received NIV (median score 16, IQR 13.25–17.75), thirteen (18.3%) were invasively ventilated (median score 16, IQR 12–18), and six examinations (8.5%) were performed in one patient receiving veno-venous ECMO therapy (median score 20.5, IQR 20–22.5); see Figure 4c. We did not witness the extubation of invasively ventilated patients in our population, as they were either referred or died before weaning from ventilation was possible. The scores of the patient receiving ECMO were significantly higher than those under invasive ventilation (*p* = 0.02), NIV (*p* = 0.03), or oxygen (*p* = 0.01); *p* > 0.05 for all other pairings. For the prediction of ECMO, a cutoff score of 20 was calculated with Sn = 0.83 and Sp = 0.92 (AUC = 0.93).

The median IL-6 was 90.4 pg/mL (IQR 28.92–358.98), and the LUS score showed a weak correlation with the IL-6 levels (ρ = 0.35, *p* < 0.001, Figure 4d). However, in 23 instances (32.4%), the patients received tocilizumab prior to LUS, corresponding to higher plasma IL-6 levels (median 689.25 pg/mL, IQR 290.75–2151.0) compared to patients naive to tocilizumab (IL-6 median 46.42 pg/mL, IQR 19.72–123.5; *p* < 0.001). Patients who received tocilizumab had higher LUS scores (median 18, IQR 14–20) than those who did not require this treatment (median 14.5, IQR 10–17, *p* < 0.001). Similarly, fifty-one (71.8%) examinations with a median score of 17 (IQR 13–19) were performed after treatment with dexamethasone, while patients naive to dexamethasone scored a median of 11.5 (IQR 5.5–16.25, *p* = 0.001).

**Figure 5:**
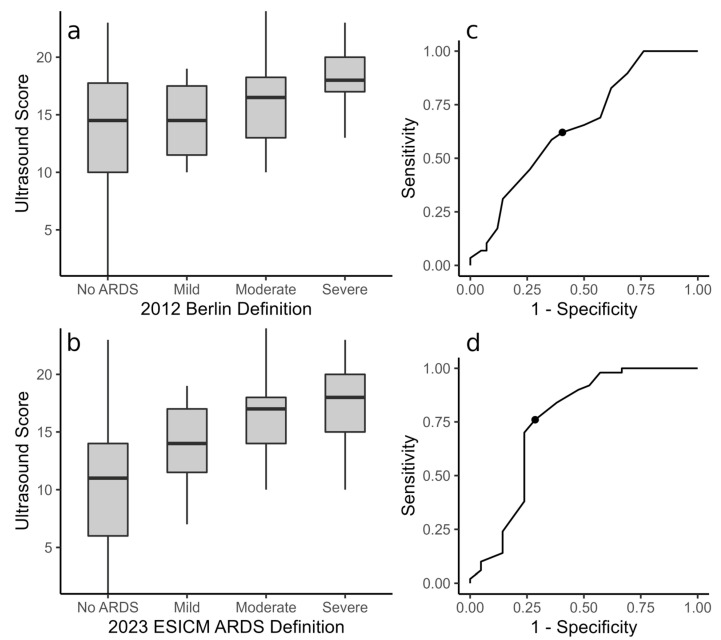
LUS score plotted against the different ARDS stages for both the (**a**) Berlin criteria [11] as well as the (**b**) revised ARDS definition from the 2023 ESICM guidelines [12]. ARDS stages cannot be discerned via LUS when using the Berlin definition (*p* = 0.1, Kruskal–Wallis test). Being able to diagnose ARDS in patients receiving HFOT (**b**) allows for a more effective distinction between disease stages (*p* = 0.002, Kruskal–Wallis test), as less invasive respiratory support is often preferred in COVID-19 patients. In ROC analysis, the calculated cutoff for ARDS when assuming the Berlin criteria (**c**) is less accurate (AUC = 0.65) than the cutoff within the revised framework in the ESICM guidelines (AUC = 0.75, (**d**)).

Over the course of the seventy-one examinations, the Berlin criteria for ARDS were positive in twenty-nine (40.8%) instances: four (5.6%) in mild, sixteen (22.5%) in moderate, and nine (12.7%) in severe ARDS (Figure 5a). Among patients without ARDS, the median score was 14.5 (IQR 10–17.75), while those with ARDS scored a median of 17 (IQR 13–19, *p* = 0.03). The scores did not differ between the ARDS stages (*p* = 0.1) when assuming the traditional Berlin definition. The post hoc analysis utilizing the renewed ARDS definition published in 2023 by the ESICM [12] allowed the allocation of patients receiving HFOT (and thus not diagnosed with ARDS according to the conventional definition) to the ARDS stages (see Figure 5b). Within this new framework, the ARDS criteria were positive in fifty (70.4%) examinations: eight (11.3%) in mild, twenty-nine (40.8%) in moderate, and thirteen (18.3%) in severe ARDS. Comparing the ARDS stages, the LUS score differed significantly when assuming the 2023 ARDS definition (*p* = 0.002). In instances when the ARDS criteria were negative, patients scored lower (median 11, IQR 6–14) than in moderate (median 17, IQR 14–19, *p* = 0.01) or severe ARDS (median 18, IQR 15–20, *p* = 0.02). The scores in mild ARDS (median 15, IQR 11.5–17) were not significantly different (*p* > 0.05 compared to severe, moderate, or no ARDS). Considering the Berlin definition, a cutoff LUS score of 16 provided Sn = 0.62 and Sp = 0.6 for the diagnosis of ARDS (AUC = 0.65, Figure 5c). A cutoff score of 14 predicts ARDS, according to the 2023 ESICM definition, with Sn = 0.76 and Sp = 0.71 (AUC = 0.75, Figure 5d).

The interrater reliability was ICC = 0.87 between the initial ultrasound examiner and the blinded evaluation by the other examiner (*p* < 0.001).

## 4. Discussion

Our results indicate a relationship between the score and the severity of pulmonary disease in this population. Moderate and severe disease as defined by the WHO [10] could be discriminated, which is similar to previous studies [14]. However, no patients with mild disease were included in this study, and no cutoff for mild disease could be calculated.

The score shows a moderate correlation with oxygenation impairment as indicated by the oxygenation (or Horovitz) index (Figure 4a). LUS primarily assesses ventilation as well as interstitial pathologies. However, COVID-19 also disrupts lung perfusion by promoting thromboembolic events and right-to-left-shunting by impairing hypoxic vasoconstriction [15,16]. Sonographic assessment of pulmonary perfusion is limited, as it can only be evaluated peripherally when consolidations are present [1,17]. Although the pulmonary perfusion deficit appears to be qualitatively representable on color Doppler ultrasonography of consolidations (see Figure 1E) [1], no quantitative sonographic measurements or diagnostic criteria are available at this point. Furthermore, not all patients with impaired pulmonary perfusion will present with conveniently located consolidations that can be utilized as ultrasound windows. LUS may, therefore, not depict all aspects of the pathophysiology of COVID-19, resulting in the broad range of the observed oxygenation indices. Differently sized consolidations will also yield the same scoring, although the limitation of the regional ventilation might be different for small subpleural or large segmental and lobar consolidations. However, implementing an overly complicated score that would appreciate all facets and configurations of possibly encountered image artifacts would be impractical for clinical use. The score is, mathematically speaking, an ordinal variable; the arbitrary intervals do not reflect identical increments in respiratory impairment. We attempted to address this by using an appropriate polyserial correlation model that accounts for correlation analysis between ordinal and continuous variables [18]. The change in score furthermore correlates to changes in oxygenation between examinations (Figure 4b). Decreasing scores might indicate a resolving pathology, as suggested by the stronger correlation between negative changes in score and corresponding improvement in oxygenation. Similarly, a study in patients receiving ECMO therapy prior to the COVID-19 pandemic found that survivors’ LUS scores decreased over the course of treatment, as opposed to non-survivors’ scores [8]. LUS could, therefore, be utilized to monitor pulmonary involvement over time, particularly due to its availability in the ICU and lack of irradiation.

Correlation with other parameters of disease progression, such as superinfection and the requirement of pharmacological treatment, further supports the relationship between the score and the severity of illness. Patients receiving dexamethasone or tocilizumab scored significantly higher than patients who did not require these therapies, which were experimental treatments at the time of this investigation and were employed in critically ill patients with COVID-19 [19,20]. Although the LUS score appears to reflect higher serum IL-6 in correlation analysis, this finding may be influenced by the treatment the patients received; statistical analysis might be distorted when sicker patients requiring tocilizumab (which might influence serum IL-6 [21]) present with increased IL-6 levels because of it. However, we also cannot rule out that patients who required tocilizumab had higher IL-6 levels independently of their treatment, as they had more severe illness warranting the use of this therapy.

The Berlin definition, consisting of acute onset (within 7 days) of lung injury with bilateral pulmonary opacities not explained by cardiac disease, impaired oxygenation, and ventilation with a PEEP of at least 5 mbar, is widely used to classify ARDS [11]. Although patients who fulfilled the Berlin definition did have higher scores than those who did not, the largely overlapping scores (Figure 5a) render it difficult to predict the diagnosis based on LUS alone. Conventionally, management of ARDS aims to treat the underlying cause while providing mechanical ventilation with PEEP [11,22]. However, in the care of lung injury due to COVID-19, awake proning and less invasive respiratory support are favored [23,24]. Therefore, we saw patients not formally meeting the Berlin definition (as no PEEP was applied), but with severely impaired oxygenation. The 2023 amendment by the ESICM to the ARDS definition, however, allows for the diagnosis of ARDS in patients receiving HFOT [12], and LUS discerns ARDS in post hoc analyses incorporating the current definition (Figure 5). As LUS scores still overlap between the groups, mild ARDS cannot be discerned from cases without ARDS or moderate and severe ARDS; however, patients with moderate and severe ARDS score significantly higher than those not meeting the ARDS definition. The cutoff score of 14 discriminates ARDS, according to the 2023 revised ESICM definition, with more reasonable sensitivity and specificity in our population compared to the cutoff score of 16 for the Berlin definition (Figure 5c,d). These findings contrast the results in ARDS patients before the COVID-19 pandemic. A study prior to the pandemic identified a cutoff of 5 for the diagnosis of ARDS using a similar scoring system [6], while the majority of patients in our study scored considerably higher than that even in the absence of ARDS, regardless of the definition considered. Presumably, the more conventional treatment strategies prior to the pandemic, including early intubation and mechanical ventilation, allowed for an earlier diagnosis of ARDS and, therefore, stronger discriminatory power [6]. Similar to this study, the score could not distinguish between the different stages of ARDS in our population either. A meta-analysis of previously published studies comparing the LUS score to the severity of COVID-19 found a broad range for different stages of illness as defined by the WHO, as well [14]. However, our present study also investigated the correlation of the score to individual markers of disease severity, such as oxygenation index, respiratory support, plasma IL-6, and pharmacological treatment, in addition to the composite (WHO and ARDS) classifications.

The calculated cutoff values are not generalizable due to the small number of examinations that were analyzed. Keeping in mind that a single diagnostic parameter is usually insufficient to predict mortality or the diagnosis of ARDS, the significantly higher scores in severe COVID-19, ARDS, and in non-survivors may be indicative of an underlying statistical effect. The cutoffs may at least help to identify patients at low risk, as suggested by the high sensitivity in predicting severe disease and mortality. However, the low specificity for the mortality cutoff renders the prediction of patients at high risk of a fatal outcome much more challenging. All examinations under ECMO therapy consistently yielded similarly high scores as in previous studies [8], and future research into the prediction of extracorporeal oxygenation in ARDS could be a promising application of the LUS score. The requirement for ECMO cannot be predicted from our results due to the low number of examinations in only one patient of our study population who received ECMO (see Figure 3).

Since the primary examiner could not be blinded to the patients, the LUS examination results may have been biased according to the patients’ clinical presentation. The blinded secondary evaluation, however, showed a high level of agreement with ICC = 0.87, which can be considered an excellent agreement [25], emphasizing the objectiveness of the score. This finding of high interrater reliability furthermore reduces the risk there was observational bias from the primary examiner who, consciously or not, witnessed other indicators of disease severity during the examination. Previous studies also demonstrated a strong correlation between the sonographic aeration score and computed tomography (CT) in COVID-19 [26], suggesting a robust agreement among imaging modalities. However, as LUS only assesses pulmonary tissue adjacent to the pleura, it cannot fully replace CT in pulmonary imaging.

Repeating examinations in the same patients may have disproportionately weighted the LUS results of sicker patients in the analysis, which could impair its validity. Furthermore, we chose a scoring system that does not assess posterior lung regions; as previous studies demonstrated the high prevalence of sonographic abnormalities in the dorsobasal lungs of critically ill patients, these could adversely influence the false positive rate and noise-to-signal ratio in interpreting the LUS findings [6,27]. Additionally, the comparison with previous studies on LUS scores is difficult, as the scoring system and number of views differ among published articles in the past [6,7,8,14].

Although our observations suggest the usefulness of LUS for monitoring patients in the critical care setting over time, the relevance is limited due to the sample size and possible bias towards sicker patients. Distinct advantages of LUS are its bedside application, avoiding transport of critically ill patients, and the lack of irradiation. The score furthermore appears to reflect trends in oxygenation, and the observed cutoff values may assist with risk stratification regarding which patients face higher mortality. The values we calculated cannot be generalized to larger populations, and future research is necessary to evaluate the feasibility of these cutoffs. Our findings furthermore underline the broad range of COVID-19-associated lung injuries and consecutive differences from studies in pulmonary disease prior to the pandemic.

## Data Availability

As agreed upon with the ethics committee, patient data will not be publicly available.
